# Anticipating changes in wildlife habitat induced by private forest owners’ adaptation to climate change and carbon policy

**DOI:** 10.1371/journal.pone.0230525

**Published:** 2020-04-02

**Authors:** Yukiko Hashida, John Withey, David J. Lewis, Tara Newman, Jeffrey D. Kline

**Affiliations:** 1 Department of Agricultural and Applied Economics, University of Georgia, Athens, Georgia, United States of America; 2 Graduate Program on the Environment, Evergreen State College, Olympia, Washington, United States of America; 3 Department of Applied Economics, Oregon State University, Corvallis, Oregon, United States of America; 4 U.S. Department of Agriculture, Forest Service, Pacific Northwest Research Station, Corvallis, Oregon, United States of America; University of Nevada, Reno, UNITED STATES

## Abstract

Conserving forests to provide ecosystem services and biodiversity will be a key environmental challenge as society strives to adapt to climate change. The ecosystem services and biodiversity that forests provide will be influenced by the behaviors of numerous individual private landowners as they alter their use of forests in response to climate change and any future carbon pricing policies that emerge. We evaluated the impact of forest landowners’ likely adaptation behaviors on potential habitat for 35 terrestrial, forest-dependent vertebrates across three U.S. Pacific states. In particular, we couple a previously estimated empirical-economic model of forest management with spatially explicit species’ range and habitat associations to quantify the effects of adaptation to climate change and carbon pricing on potential habitat for our focal species (amphibians, birds and mammals) drawn from state agency lists of species of conservation concern. We show that both climate change and carbon pricing policies would likely encourage adaptation away from currently prevalent coniferous forest types, such as Douglas-fir, largely through harvest and planting decisions. This would reduce potential habitat for a majority of the focal species we studied across all three vertebrate taxa. The total anticipated habitat loss for amphibians, birds and mammals considered species of state concern would exceed total habitat gained, and the net loss in habitat per decade would accelerate over time. Carbon payments to forest landowners likely would lead to unintended localized habitat losses especially in Douglas-fir dominant forest types, and encourage more hardwoods on private forest lands. Our study highlights potential tradeoffs that could arise from pricing one ecosystem service (e.g., carbon) while leaving others (e.g., wildlife habitat) unpriced. Our study demonstrates the importance of anticipating potential changes in ecosystem services and biodiversity resulting from forest landowners’ climate adaptation behavior and accounting for a broader set of environmental benefits and costs when designing policies to address climate change.

## Introduction

Climate change has emerged as a widely acknowledged factor in biodiversity loss [1]. One pathway through which climate change could affect biodiversity is through the adaptation decisions of forest landowners. By altering productivity of commercially valuable tree species such as Douglas-fir in the Pacific states of the U.S. [2] climate change creates incentives for landowners to switch the types of trees they replant, thereby creating landscape changes to the composition of different types of forests [3]. Changes in the composition of forest landscapes alters habitat for wildlife, particularly those species that are specialized to specific types of forests. The extent to which climate adaptation goals meant to maximize the productive value of land for timber producers can generate broader social costs through changes in habitat for sensitive wildlife is not currently understood.

Carbon pricing policies—penalizing carbon emissions and rewarding carbon storage—are widely advocated as a way to mitigate the negative impacts associated with climate change [4]. In managed forests, carbon pricing policies compensate forest landowners for increasing carbon storage, whether through reduced harvesting, increased tree planting, or other forest management activities. For example, carbon pricing can influence replanting decisions since tree species that sequester carbon at higher rates may become more profitable [5]. However, any pricing policy that targets one particular ecosystem service—carbon storage in this case—has the potential to incentivize landowners to choose management strategies that ignore the provision of other ecosystem services that remain unpriced (e.g., habitat for a diverse suite of wildlife species, or water quality). Therefore, carbon pricing policies potentially can affect biodiversity by changing land management incentives in ways that alter species’ habitats. The extent to which carbon pricing policies influence ecological values and enhance overall social welfare ultimately depends on how other unpriced ecosystem services are affected by resulting forest management changes, as well as the magnitude of social values associated with other affected ecosystem services.

Anticipating climate and policy impacts on the species composition of forest landscapes—both vegetation and wildlife—calls for characterizing disturbance regimes that induce forest change. Forest disturbance can arise from natural processes (e.g., wildfire, insects and disease outbreaks) or by direct human manipulation (e.g., timber harvest), all of which can be considered ‘pulse’ effect disturbances [6]. Climate change can influence forest disturbance as a ‘press’ or chronic effect, by altering wildfire likelihood and severity [7], and by altering the growth of commercially valuable tree species [2] and other vegetation. Forest landowner adaptation can both influence, and be influenced by, forest disturbance, whether through fire management, such as thinning or controlled burns, or harvest and replanting of different species [8]. A recent global analysis classifying factors that influence forest disturbance found that harvest activity accounts for the vast majority of forest disturbance in the conterminous U.S., Europe, southern Canada, China, and Japan; while wildfire accounts for the majority of forest disturbance in northern Canada and eastern Russia [9]. In our study region of the three U.S. Pacific coast states (California, Oregon and Washington), timber harvests account for 70–87 percent of disturbance on non-federal forest lands [10,11], which make up 45 percent of Pacific coast forests. Explicitly modeling private forest landowner adaptation behavior enables anticipating how disturbance regimes might change over time in response to climate change and thus contribute to changes in forest composition and wildlife habitat.

Quantifying landscape and wildlife habitat changes is important for sustainability and conservation science because forests support biologically diverse ecosystems [12] and provide socially valuable ecosystem services [13]. Since the majority of the world’s temperate and boreal forest land is actively managed to provide market goods [14], the potential for managed forest lands to provide wildlife habitat [15] in support of biodiversity depends on the choices of forest managers—when and how intensively they harvest, how intensively they thin stands for fire management or other objectives, and what tree species they replant or regenerate after harvest. Many wildlife species remain unprotected as they occupy lands outside of protected areas and many critical threats to species do not respect protected-area boundaries, including climate change [16]. As the state of terrestrial biodiversity depends largely on the effect of human use of unprotected lands [17], it is important to understand whether working forestland can support biodiversity while satisfying human needs in the presence of both climate change and climate change mitigation policy.

In the natural science literature, many studies have focused on projecting species’ range shifts in response to climatic changes (e.g., via bioclimatic envelope modeling [18–20]), though such efforts typically have not modeled landowner adaptation to climate change. Economics studies have examined the effects of carbon pricing on landscape change in temperate forests, but typically have not accounted for the likelihood that climate change may alter the productive capacity of land [21–25]. Other studies have examined the influence of carbon pricing policies on land-use change into and out of forests [21,24,25], but have not explicitly accounted for landowners’ climate change adaptation behaviors, nor modeled changes within existing forests. Finally, some studies have examined biodiversity impacts from deforestation and forest degradation (REDD) policies in tropical forests [26], which are typically assumed to have positive impacts for biodiversity [27], but they have not simultaneously addressed adaptation to climate change.

In this paper, we provide to our knowledge the first empirical economic model of forest management adaptation to climate change and carbon pricing, integrated with spatially explicit data on potential habitat for 35 vertebrate species of concern, in order to quantify the combined effects of climate change and carbon pricing on forest-dependent wildlife. We focus on climate and policy effects that are directly induced through management choices by private landowners on non-federal forests across the three Pacific coast states. We model climate adaptation by landowners with micro-econometric methods to estimate how forest management—including harvest and replanting—as influenced by climatic, biophysical, and economic variables at fine spatial scales. We integrate the plot-level econometric model with species-specific habitat associations to simulate changes in potential wildlife habitat for conservation-sensitive vertebrate species on non-federal forest land across the Pacific states. Each vertebrate species (amphibians, birds and mammals) has been listed as a species of concern by at least one of the three Pacific coast states, and all are habitat specialists requiring specific forest types (e.g., Douglas-fir, ponderosa pine, hardwoods, etc.) for prime breeding habitat. Such habitat needs make these species sensitive to replanting decisions that alter the mix of forest types available as future potential habitat. Carbon pricing is a relevant policy for the Pacific states, as California currently operates a state-wide carbon cap-and-trade program, and similar systems are under consideration in Washington and Oregon. Our integration of economic-ecological models complements other efforts that study species’ vulnerability to climate change, by emphasizing the importance of modeling human resource management decisions.

## Methods

### Potential forest habitat for wildlife species

**Fig 1:**
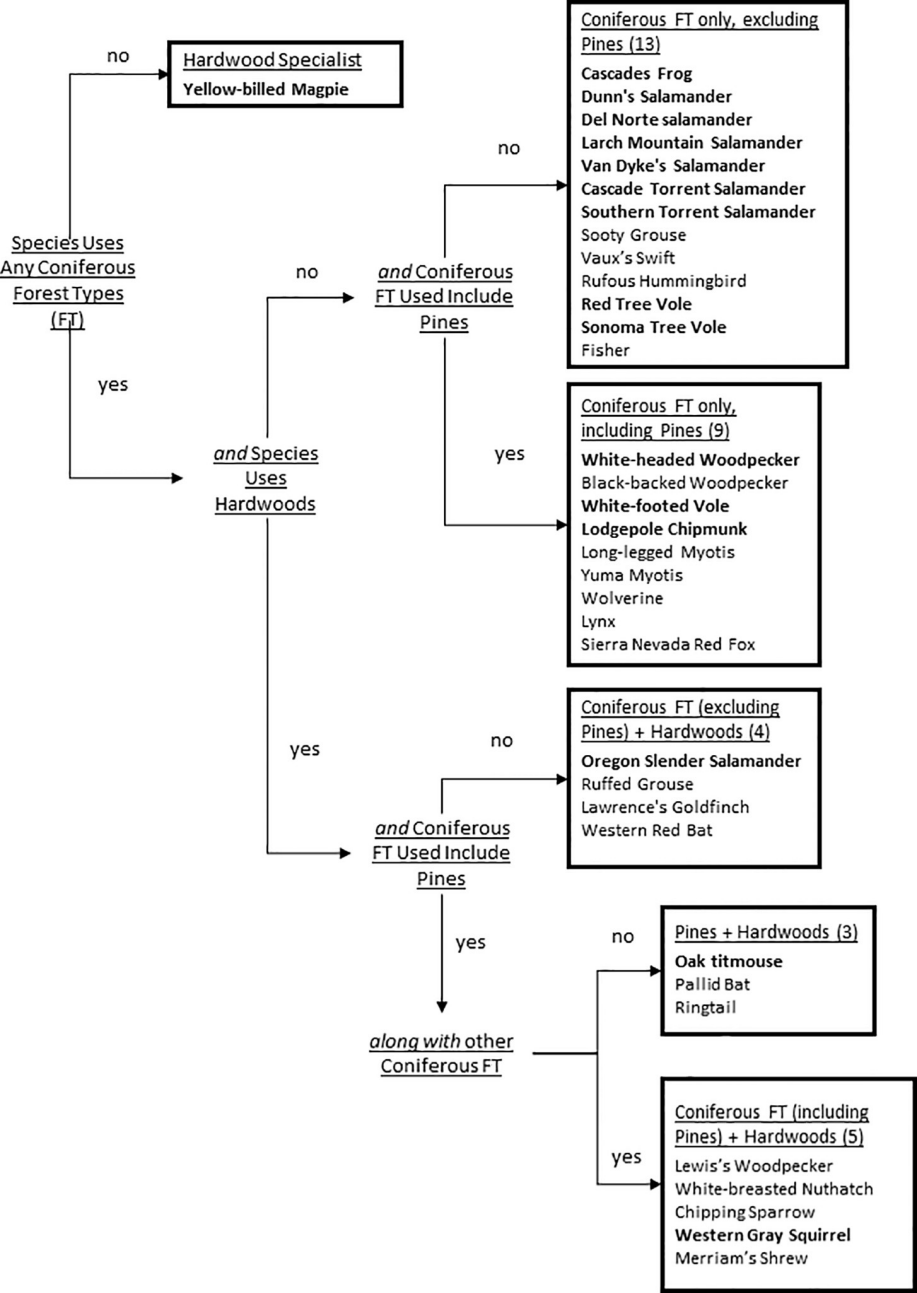
Classification of wildlife species by forest types used/not used (full details in S1 Table; here ‘Coniferous FT’ include Douglas-fir, fir/spruce, and hemlock/sitka spruce groups as defined by FIA forest types). Species names in BOLD indicate ≥50% of that species’ range lies within our three U.S. Pacific coast states study area.

We created a list of vertebrate species considered of conservation concern by state agencies of California, Oregon, and Washington [28–30]. We calculated potential forest habitat for species of state conservation concern based on the following criteria: 1) species associated with forest habitats (i.e., not primarily open, grassland, or wetlands-dependent species); 2) associated with only some and not all FIA forest types (i.e., not a forest generalist); 3) not extremely range limited (>500 FIA plots within the species’ range); 4) not an old-growth specialist (since harvested forestlands do not typically reach mature age classes). Filtering the state lists by these criteria resulted in 35 species which included amphibians (*n* = 8), birds (*n* = 12), and mammals (*n* = 15); no reptiles on state agency lists met all four criteria. We obtained range maps and distribution models for these species, and the Protected Areas Database of the U.S. (PAD-US), from the USGS National Gap Analysis Program [31,32]. We also calculated: the proportion of each species’ range as a whole that was overlapped by our 3-state study area; and the proportion of each species’ range, clipped to our 3-state study area, that was overlapped by protected areas (using GAP codes 1 and 2 in the PAD-US layer [31]; S1 Table). In order to determine which forest types we would consider as potential breeding habitat for a given species, we used state wildlife agency reports and habitat associations described in the NatureServe online database (available from http://explorer.natureserve.org/index.htm). Based on those associations, a binary value was assigned to each forest type for each species (1 = used, 0 = not typically used, Fig 1, S1 Table).

### Econometric-based landscape simulation of potential forest habitat for wildlife

We use Hashida and Lewis’ [3] econometric model of forest management to simulate changes in the composition of potential forest habitat for wildlife under alternative climate change and carbon pricing scenarios. The econometric model is estimated from the U.S.D.A. Forest Service’s Forest Inventory and Analysis (FIA) plot-level database describing individual private forest landowners’ management choices (harvest, harvest intensity, and replanting) and natural disturbance (fire, insects, and disease). The model uses observed management choices to quantify the influence of timber prices, land quality, and climate on the probability of choosing to harvest and replant with different forest types, and the probability and intensity of natural disturbances such as wildfire. The climate variables we included in our analysis include total precipitation and mean temperature during the growing season, maximum temperature in August, and minimum temperature in December. Growing season months are those that have growing degree days above 10° Celsius (50°F), which are determined at a regional level that represents varying climate zones. All climate variables are based on normal monthly data from the Parameter-elevation Regressions on Independent Slopes Model (PRISM) over a 30-year period between 1981 and 2010. The estimation sample includes over 6,800 private and state-owned forest plots in Oregon, Washington, and California. The study region has considerable climate variation and corresponding variation in tree species types (S3a Fig). Each plot is assigned a set of forest types available for replanting based on plant viability scores for each location [33].

**Fig 2:**
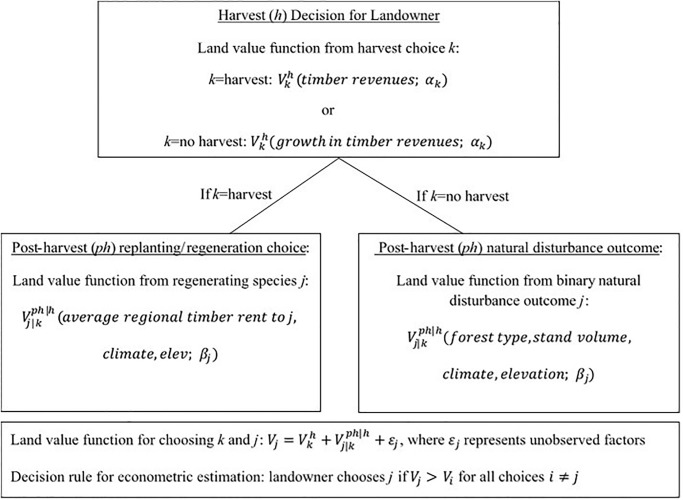
Schematic of econometric model of forest management. Estimation is based on the principle that landowners choose forest management to maximize their economic land value. Parameter vectors *α*, *β* are estimated in Hashida and Lewis [3].

Fig 2 presents a visual schematic of the econometric model. The model’s estimated parameters reflect landowners’ preferences for different management options as revealed by their actual management choices in response to factors such as prices, tree growth, climate, and elevation. The first decision a landowner makes is whether to harvest as a clear-cut or partial-cut, or not harvest and let the trees continue to grow. Each harvest choice affects a landowner’s economic land value, and the land value function of harvest choice *k* is denoted as $V_{k}^{h}$. The land value function from harvest $V_{k}^{h}$ is a function of revenues a landowner receives from harvest, and the potential growth in harvest revenues if a landowner refrains from harvest and lets the stand grow (Fig 2). If a landowner harvests, the next choice is whether to replant/re-generate post-harvest in one of six primary forest types—Douglas-fir, fir/spruce/mountain hemlock, hemlock/sitka spruce, ponderosa pine, other softwoods, or hardwoods. These forest types are groupings defined by the U.S.D.A. Forest Service FIA Program. Since there is a lack of variation needed to econometrically model landowner choices between different types of hardwoods, we grouped all hardwood tree species into one forest type.

A landowner’s economic land value depends on the replanting choice, and the land value function of the post-harvest replanting choice *j* is denoted as $V_{j|k}^{ph|h}$. The land value function from replanting species *j* is a function of the regional average annual timber rent (in dollars) for species *j*, the set of down-scaled climate variables mentioned above, and elevation (Fig 2). The rent variable measures profitability from growing species *j* and is constructed from empirical estimates of species-specific timber yield curves (volume as a function of age) for similar site classes in the region that includes the landowner’s plot, and represented as a regional average. If landowners do not harvest, their trees either continue to grow in the next time period or are affected by natural disturbance such as wildfire. The economic land value function from natural disturbance outcome *j* is a function of forest type, stand volume, climate, and elevation (Fig 2).

Combining the harvest and post-harvest decisions and outcomes, the land value function for management choice *j* is defined as $V_{j}\;=\;V_{k}^{h}+V_{j|k}^{ph|h}+\varepsilon_{j}$, where *ε*_*j*_ denotes unobservable factors specific to management choice *j*. The econometric estimation uses observed timber management choices to estimate parameters of the functions $V_{k}^{h}$ and $V_{j|k}^{ph|h}$, denoted as vectors *α*, *β* in Fig 2. Estimation is based on the discrete-choice decision rule that each landowner chooses *j* to maximize her land value, where *j* is chosen if *V*_*j*_ > *V*_*i*_ for all *j* ≠ *i*. Train [34] shows how this type of discrete-choice maximization problem generates an estimable probability that a landowner chooses management action *j* as a product of the probability of harvest action *k* multiplied by the probability of post-harvest outcome *j* conditional on choosing harvest *k*:

$$Prob\left(management\;j\right)=Prob_{k}\cdot Prob_{j|k}=\frac{\text{exp}\left(V_{k}^{h}+\lambda_{k}I_{k}\right)}{\sum_{k=1}^{K}\text{exp}\left(V_{k}^{h}+\lambda_{k}I_{k}\right)}\cdot \frac{\text{exp}\left(V_{j}^{ph|h}/\lambda_{k}\right)}{\sum_{j=1}^{J}\text{exp}\left(V_{j}^{ph|h}/\lambda_{k}\right)}$$ (1)

The term *λ*_*k*_ is known as the inclusive value term in this nested logit model, and represents the maximum of $V_{j|k}^{ph|h}$ [34]. Embedded in (1) are plot-level probabilities that each landowner harvests forestland in clear-cut or partial-cut, or leaves the stand undisturbed to grow (*Prob*_*k*_). Conditional on the landowner harvesting the land, Eq (1) embeds plot-level probabilities of the forest type that the landowner chooses to replant or regenerate on her land (*Prob*_*j*|*k*_). Conditional on not harvesting their land, Eq (1) embeds a plot-level probability of natural disturbance (*Prob*_*j*|*k*_). Climate affects *Prob*_*k*_ and *Prob*_*j*|*k*_, and hence, changes in climate will induce changes in *Prob*_*k*_ and *Prob*_*j*|*k*_. Note that the above development suppresses notation for each plot and time period for simplicity—see S1 Text and [3] for a more precise mathematical development.

Each landscape simulation uses the econometrically estimated probabilities of discrete forest management choices and natural disturbance outcomes as a set of decision rules and projects the time-path of landscape change and corresponding potential habitat change for wildlife. Beginning with the current stock of forest habitat on the landscape, the estimated probabilities of each forest management decision are calculated in 10-year time steps up to 2090 in response to exogenous changes in climate (RCP 8.5), global timber prices [35], and net primary productivity (NPP) of forests [36]. Timber rotation lengths, natural disturbance outcomes, and replanting outcomes are stochastic and heterogeneous across plots.

We consider three scenarios for this paper. The *baseline* scenario assumes no climate change or carbon pricing. In a *climate change only* scenario, future climates change according to the RCP 8.5 scenario, which is consistent with current emissions trajectories [37,38]. Future climate regimes are derived from the U.S. National Center for Atmospheric Research (NCAR) Community Climate System Model (CCSM) 4. In a *climate change + carbon pricing* scenario, we also determined the payment to each landowner as a rent for the carbon sequestration ability of their land combined with a tax for any carbon released at harvest [39,40]. We assume that a carbon price starts at $15/ton in 2020, rises to $50 in 2050, and again to $80 in 2080. To examine the sensitivity of our results to carbon price inputs, we also used a ‘low carbon price’ scenario ($15/ton in 2020, up to $30/ton in 2050, then to $50/ton in 2080) and a ‘high carbon price’ scenario ($30/ton in 2020, up to $60/ton in 2050, then to $100/ton in 2080). Carbon sequestration on each plot is derived by estimating carbon yield curves from the FIA data, whereby each carbon yield curve represents tons of carbon sequestered as a function of the age of the tree stand. By differentially augmenting the rents received from timber across forest types, the carbon pricing scheme effectively pays forest landowners for providing two ecosystem services—timber and carbon. For each scenario (baseline, climate change only, and three versions of the climate change + carbon pricing), 1,000 individual simulations were run to represent the variability in projected changes.

Based on each of the 35 species’ individual forest type use (S1 Table), we calculated the proportion of forest plots inside each species’ range that would be a potentially suitable forest type. We also kept track of this separately for each ecoregion inside a species’ range (creating multiple species by ecoregion combinations, as long as an individual ecoregion had a minimum of 50 FIA plots inside the species’ range). We did this for each time step and produced summary statistics for each scenario based on the 1,000 simulations. Results are presented based on the difference of alternative scenarios, to the baseline scenario.

We took two approaches to account for uncertainty in our estimates of potential habitat gain or loss for each species. One was to use the mean results from the low and high carbon price scenarios to bracket the mean from our main climate change + carbon pricing scenario. The second approach was based on the main finding of Hashida and Lewis [3], of landowners shifting from Douglas-fir to other species. Under each scenario we ordered the 1,000 simulations from greatest loss of Douglas-fir, to the most gain of Douglas-fir through 2100. For any given species, across its entire range we calculated the mean amount of change across all 1,000 simulations. To calculate a projection interval, we also took the 100 simulations at the two extreme ‘tails’ of the 1,000 simulations, and calculated the mean amount of potential habitat gain or loss based on 1) simulations in the ‘high tail’ of more Douglas-fir change (loss), and 2) simulations in the ‘low tail’ of less Douglas-fir loss (which for some of the 100 simulations in this tail, was a gain). These projection intervals were used to bracket the mean results for the climate change only scenario, as well as to represent uncertainty in the climate change + carbon pricing scenarios *in addition to* the variation in results due to the low and high carbon price inputs.

## Results

**Fig 3:**
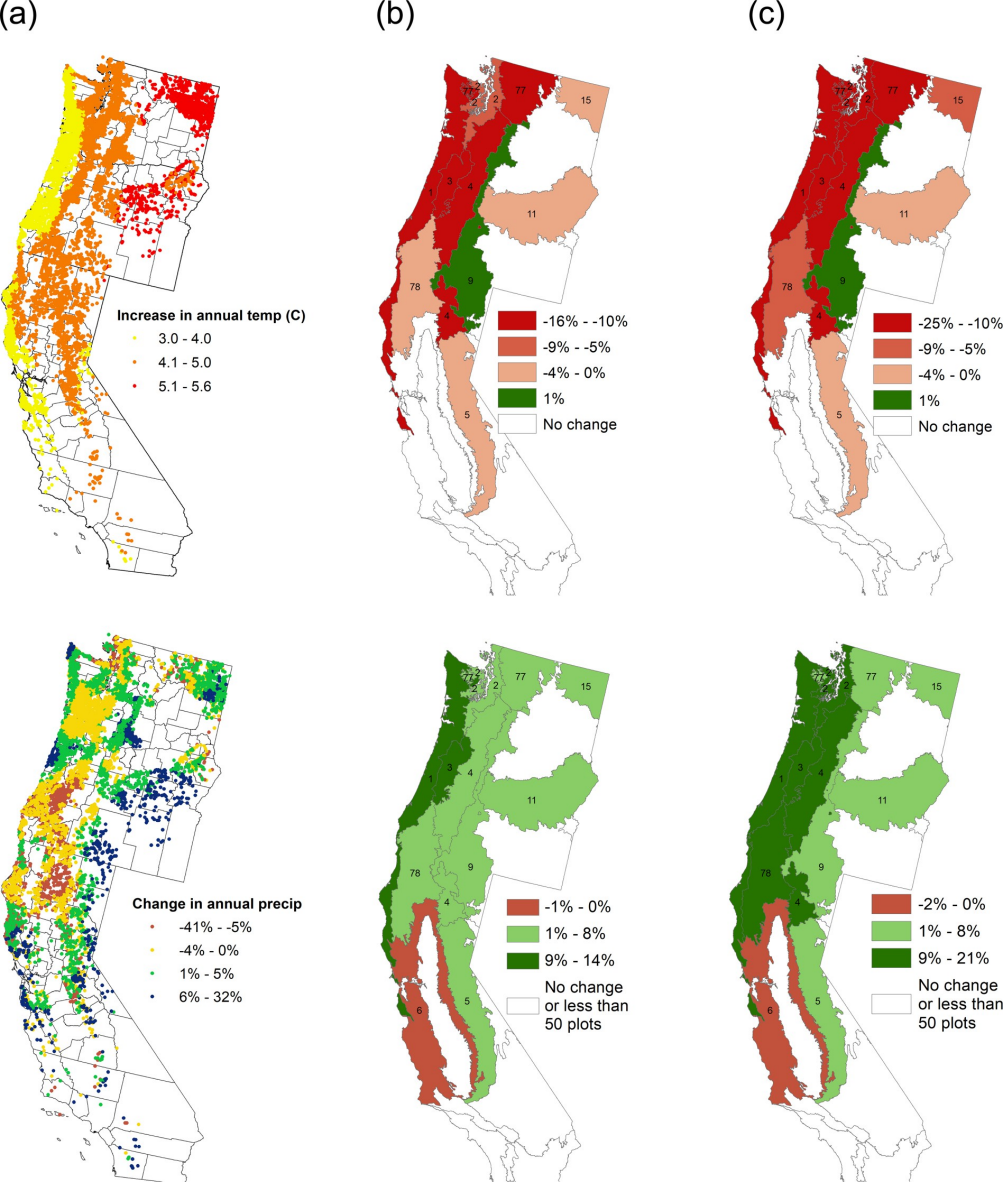
Through 2100, (a) major climatic changes, and modeled effects of (b) climate change only or (c) climate change + carbon pricing, for Douglas-fir (top) and hardwood (bottom) forest types. Dots in a. represent FIA plots (not to scale). Each number in b. and c. corresponds to level 3 ecoregions (see S6 Fig for ecoregion names). The maps were created by the authors based on the NCAR CCSM4 (a) and our simulation results (b and c) with the ArcMap software, an ArcGIS application developed by Esri [41], based on the level 3 ecoregions map from the U.S. Environmental Protection Agency (EPA) [42]. a. Projected temperature change (top) and precipitation change (bottom) under RCP 8.5. b. Percentage point difference between baseline and climate change only scenario for Douglas-fir (top) and hardwood (bottom) forest types. c. Percentage point difference between baseline and climate change + carbon pricing scenario for Douglas-fir (top) and hardwood (bottom) forest types.

We used our forest landscape simulation model to project changes in forest types on non-federal forest land from 2010 to 2100 under baseline, climate change, and climate change + carbon pricing scenarios. At the plot level, replanting choices by landowners typically are made every few decades following periodic harvests, creating a gradual adaptation response over time. Downscaled climate projections for Oregon and Washington, as well as northern California mostly anticipate warmer and drier conditions (Fig 3a), favoring hardwoods over Douglas-fir. Warmer and drier conditions favor hardwoods over Douglas-fir in the econometric model [3] because landowners who currently manage forests in warm and dry conditions (e.g. California) have been observed to be much less likely to plant Douglas-fir than landowners who currently manage forests in more temperate and wet conditions (e.g. western Oregon and Washington). The estimated econometric parameters reflect landowner responses to the climate they face, and provide a functional link between climate and the probabilities of specific management practices. For example, a 3°C increase in mean annual temperature for western Oregon and western Washington is estimated to reduce the plot-level probability of replanting Douglas-fir by approximately 30 percentage points (p<0.01) [3]. Relative to the baseline, climate change is therefore expected to increase the proportion of forest land in the hardwood forest type in the western portion of the study area, largely at the expense of the Douglas-fir forest type (Fig 3b).

Adding carbon pricing to the climate change scenario reinforces the climate change-only effect by accelerating the loss of the Douglas-fir forest type in the western portion of our study region (Fig 3c). The addition of carbon pricing would also reinforce expansion of the hardwood forest type. Our carbon price scenario assumes that landowners’ response to a hypothetical $1 carbon payment would be the same as their response to $1 in timber rents as estimated by our econometric model of timber harvest decisions. Our econometric results suggest that the average Douglas-fir plot will become less productive (with generally slower growth) under climate change in western Oregon and Washington, while the productivity of the average hardwood plot will remain largely unchanged. This results in additional carbon rents having a larger positive influence on regenerating hardwoods relative to Douglas-fir.

**Fig 4:**
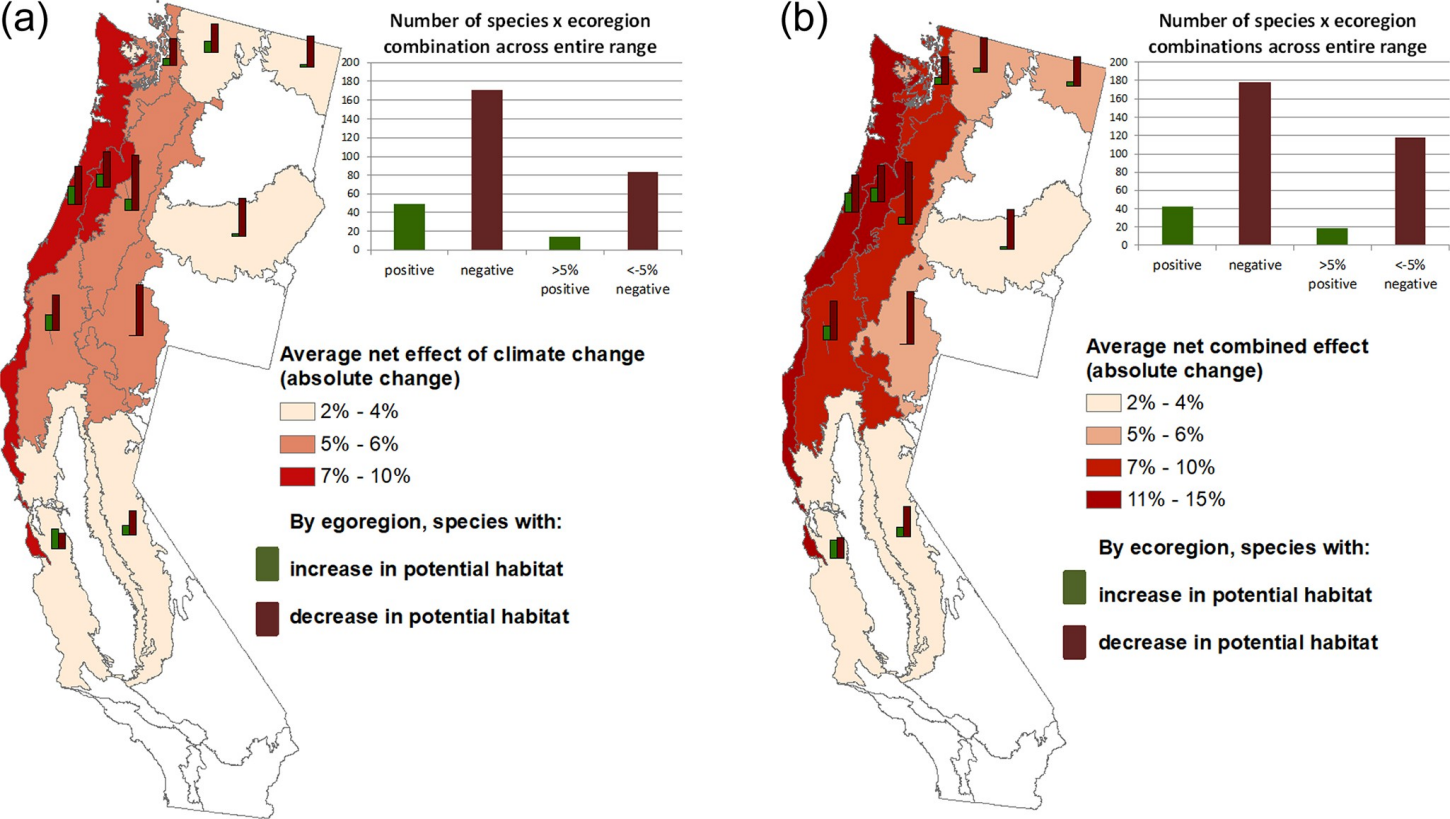
Modeled effects of climate change and carbon pricing on potential habitat of wildlife species, by ecoregion. The bar graphs within the maps show, for each ecoregion, the number of species positive change in potential habitat (green) and the number of species with negative change in potential habitat (brown). Changes in both a) and b) are relative to the baseline scenario. The bar graphs on the top right of each map show, in the 2 left bars, the total number of positive or negative species-ecoregion changes (the sum of all the bar graphs within each map). The two right bars show a subset of the species-ecoregion changes: “>5% positive” includes the species in an ecoregion that gain >5% potential habitat by 2100 relative to the baseline, and “>5% negative” includes the species in an ecoregion that lose >5% potential habitat by 2100 relative to the baseline (5% was chosen to represent changes of more practical significance). The maps and bar graphs were created by the authors with the ArcMap software [41]. a. Effects of climate change. b. Effects of climate change + carbon pricing.

Combining our landscape projections with detailed habitat associations, we projected changes in potential habitat (forest types suitable for each species; S1 Table) within the current ranges of 35 wildlife species already identified by state agencies as species of concern. Our projections suggest that climate adaptation by forest landowners will result in many more species experiencing significant reductions, rather than gains, in potential habitat (Fig 4a, Table 1), relative to the baseline. Across species-ecoregion combinations, potential habitat is projected to decline for 171 combinations and increase for 49 combinations. Potential habitat losses are projected for large numbers of species in coastal ecoregions currently dominated by Douglas-fir and spruce forest types. These losses are borne by species that depend on such forests, and primarily caused by the projected shift from conifers (excepting ponderosa pine) to hardwoods following harvest (Fig 3). Significant losses are also projected in eastern ecoregions for wildlife species that do not typically use hardwoods but specialize in coniferous forest types—some of which use ponderosa pine (e.g., Yuma myotis, Sierra Nevada red fox), others of which do not (e.g., Cascades frog, Vaux’s swift, fisher).

**Table 1:** Forest-dependent terrestrial vertebrates (*n* = 35) in taxonomic order, with percent change in potential habitat presented for our scenarios relative to the baseline. ‘Mean % change’ is the change, through 2100, in the number of FIA plots considered suitable potential habitat (based on associations shown in S1 Table), across all 1000 simulations in each main scenario relative to the baseline, across a species’ range, In the Climate Change Only section, results presented in columns for ‘High tail’ and ‘Low tail’ of DF (Douglas-fir) change represent our prediction interval around the mean (see Methods). In the Carbon Pricing + Climate Change section, ‘High CP’ represents the mean % change from the high carbon price version of that scenario, with ‘(+high DF)’ the additional prediction interval from the high tail of DF change. ‘Low CP’ represents the mean % change from the low carbon price version of that scenario, with ‘(+low DF)’ the additional prediction interval from the low tail of DF change (see Methods for additional description).

Common name	Scientific name	Climate Change Only			Carbon pricing + Climate Change		
		High tail of more DF change	Mean % change	Low tail of more DF change	High CP (+high DF)	Mean % change	Low CP (+low DF)
**Amphibians**							
Cascades frog	*Rana cascadae*	-13.0	-7.0	-2.0	-11.3 (-17.3)	-10.7	-9.3 (-4.3)
Oregon slender salamander	*Batrachoseps wrighti*	-9.4	-8.1	-7.0	-7.4 (-8.7)	-7.4	-8.0 (-6.9)
Dunn’s salamander	*Plethodon dunni*	-30.5	-17.7	-8.0	-28.3 (-41.1)	-26.6	-23.1 (-13.4)
Del Norte salamander	*Plethodon elongatus*	-11.7	-3.0 ^ns^	+7.8	-10.0 (-18.7)	-8.5 ^ns^	-7.0 ^ns^ (+3.9)
Larch Mountain salamander	*Plethodon larselli*	-13.3	-6.0 ^ns^	+0.7	-11.5 (-18.8)	-9.7	-8.6 (-1.9)
Van Dyke’s salamander	*Plethodon vandykei*	-23.8	-13.2	-5.6	-23.0 (-33.7)	-21.1	-18.1 (-10.5)
Cascade torrent salamander	*Rhyacotriton cascadae*	-19.5	-10.3	-3.2	-20.2 (-29.4)	-18.1	-15.0 (-7.9)
Southern torrent salamander	*Rhyacotriton variegatus*	-23.9	-14.2	-5.8	-23.5 (-33.2)	-21.6	-18.8 (-10.4)
**Birds**							
Ruffed grouse	*Bonasa umbellus*	-0.8	-0.7	-0.4	-0.4 (-0.5)	-0.5	-0.6 (-0.3)
Sooty grouse	*Dendragapus fuliginosus*	-23.5	-13.2	-5.1	-21.9 (-32.2)	-20.2	-17.7 (-9.6)
Vaux’s swift	*Chaetura vauxi*	-15.7	-9.0	-3.4	-14.8 (-21.5)	-13.8	-11.9 (-6.3)
Rufous hummingbird	*Selasphorus rufus*	-15.9	-9.2	-3.6	-15.1 (-21.8)	-14.0	-12.2 (-6.5)
Lewis’s woodpecker	*Melanerpes lewis*	-2.2	-1.7	-1.3	-1.6 (-2.1)	-1.6	-1.7 (-1.3)
White-headed woodpecker	*Picoides albolarvatus*	-4.0	-2.1	-0.8	-4.9 (-6.7)	-4.1	-3.3 (-2.0)
Black-backed woodpecker	*Picoides arcticus*	-3.5	-1.9	-0.7	-4.6 (-6.2)	-3.9	-3.1 (-1.9)
Yellow-billed magpie	*Pica nuttalli*	+2.1	+1.8	+1.6	+2.3 (+2.7)	+1.4	+1.6 (+1.5)
Oak titmouse	*Baeolophus inornatus*	+3.6	+2.1	+0.2	+4.3 (+5.7)	+3.5	+2.8 (+0.8)
White-breasted nuthatch	*Sitta carolinensis*	-1.9	-1.3	-0.8	-1.0 (-1.6)	-1.0	-1.2 (-0.7)
Chipping sparrow	*Spizella passerina*	-0.3	0.0 ^ns^	+0.6	+0.6 (+0.3)	+0.5	+0.3 (+0.8)
Lawrence’s goldfinch	*Spinus lawrencei*	+0.7	+0.2 ^ns^	-0.3	+1.7 (+2.2)	+0.2 ^ns^	+0.1^ns^ (-0.4)
**Mammals**							
White-footed vole	*Arborimus albipes*	-24.5	-13.2	-4.8	-22.3 (-33.6)	-20.6	-17.7 (-9.3)
Red tree vole	*Arborimus longicaudus*	-31.1	-18.4	-8.2	-28.5 (-41.3)	-26.8	-23.5 (-13.4)
Sonoma tree vole	*Arborimus pomo*	-20.0	-12.7	-2.2	-42.6 (-49.9)	-33.4	-25.1 (-14.6)
Lodgepole chipmunk	*Neotamias speciosus*	-2.4	-1.2	-0.7	-1.6 (-2.9)	-0.3	-0.7 (-0.3)
Western gray squirrel	*Sciurus griseus*	-2.8	-2.1	-1.6	-2.4 (-3.1)	-2.2	-2.3 (-1.8)
Merriam’s shrew	*Sorex merriami*	-3.5	-3.1	-2.8	-1.8 (-2.2)	-2.3	-2.6 (-2.2)
Pallid bat	*Antrozous pallidus*	+14.3	+8.0	+2.4	+15.7 (+22.0)	+13.9	+11.5 (+5.9)
Western red bat	*Lasiurus blossevillii*	-1.6	-1.4	-0.9	-1.4 (-1.6)	-1.6	-1.5 (-1.0)
Long-legged myotis	*Myotis volans*	-10.9	-5.9	-2.1	-11.1 (-16.2)	-9.9	-8.3 (-4.5)
Yuma myotis	*Myotis yumanensis*	-15.5	-9.0	-4.2	-14.8 (-21.2)	-13.3	-11.8 (-6.9)
Ringtail	*Bassariscus astutus*	+10.5	+6.7	+2.6	+11.2 (+15.0)	+10.1	+8.6 (+4.5)
Wolverine	*Gulo gulo*	-3.0	-1.8	-0.8	-5.0 (-6.2)	-4.1	-3.2 (-2.2)
Lynx	*Lynx canadensis*	-2.5	-1.9	-1.3	-5.5 (-6.1)	-4.7	-3.6 (-2.9)
Fisher	*Pekania pennanti*	-13.4	-8.0	-2.9	-13.2 (-18.5)	-12.2	-10.4 (-5.3)
Sierra Nevada red fox	*Vulpes vulpes necator*	+1.4	+1.0 ^ns^	-0.1	+1.3 (+1.7)	+1.0 ^ns^	+1.1 ^ns^ (0.0)

We examined the combined effect of climate change and carbon pricing by subtracting the baseline scenario from the carbon pricing scenario (Fig 4b, Table 1). Carbon pricing is projected to increase forest landowners’ adaptation away from coniferous forests, in favor of hardwoods. Carbon pricing also would increase the number of species-ecoregion combinations that would see larger than 5% reductions in their potential habitat and accelerates the reduction of potential habitat for several species.

**Fig 5:**
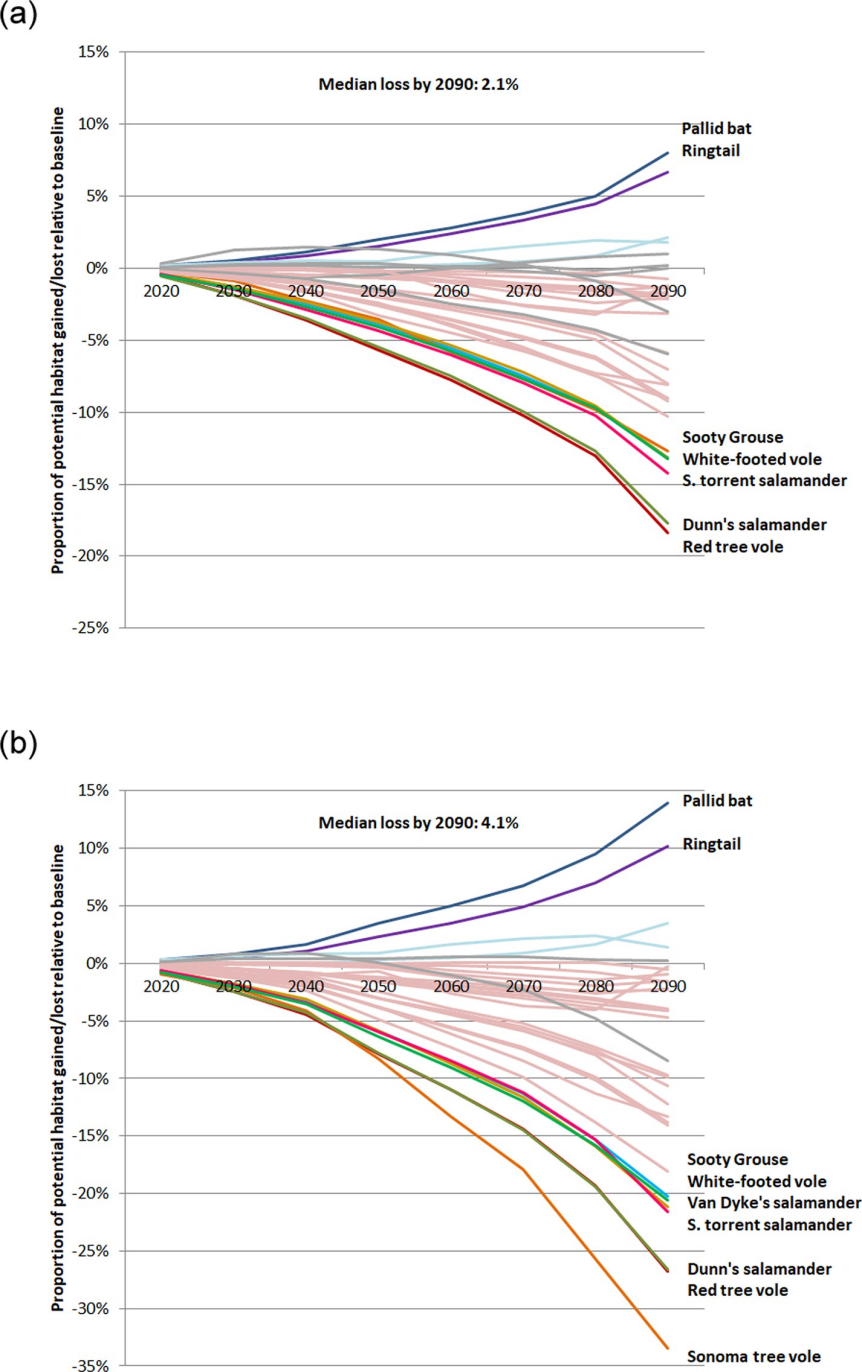
The proportion of FIA plots gained or lost within a species’ entire range that is potential habitat, under the (a) climate change only scenario and (b) climate change + carbon pricing scenario, relative to the baseline scenario. We list selected species’ names corresponding to solid colored lines on the right side of each plot, from largest gains (top) to largest losses (bottom). Other species with significant projected gains are represented by a light aqua line, species with significant losses by a light red line, and species with non-significant changes by a grey line (see Table for % change values, including prediction intervals, by scenario, for each species). a. Effects of climate change. b. Effects of climate change + carbon pricing.

Species projected to experience the largest losses in potential habitat (of 8% or more under the climate change only scenario, and 12% or more under climate change + carbon pricing) include amphibians (Dunn’s salamander, Van Dyke’s salamander, and both torrent salamanders) three birds (sooty grouse, , rufous hummingbird, and Vaux’s swift), all three voles (red tree vole, white-footed vole, and Sonoma tree vole), one bat (Yuma myotis), and one carnivorous mammal (fisher; Fig 5; Table 1). None of these 12 species uses hardwoods, and only two use ponderosa pine—the two forest types expected to expand under the climate change and carbon pricing scenarios [3]. As a group, amphibians of concern are the most likely to have significant habitat losses under all scenarios (Table 1), reflecting their dependence on forest types that are typically wetter (e.g. using Douglas-fir but not ponderosa pine, Fig 1, S1 Table). Four species were projected to gain potential habitat under our scenarios: one hardwood forest type specialist (yellow-billed magpie), and specialists to both the hardwoods and ponderosa pine forest types (oak titmouse, ringtail, and pallid bat; Figs 1 and 5, Table 1). Lines with kinks from 2080 to 2090 are two range-limited species (lodgepole chipmunk and Del Norte salamander) with just 6.6% and 4.3% of total FIA plots, which could explain these relatively abrupt changes.

Our results suggest that wildlife species across multiple taxa that depend on coniferous forests (other than ponderosa pine) largely will lose habitat, with greater magnitudes of loss under the carbon pricing scenario. For some species, in particular some salamanders and the tree voles (*Arborimus* spp.), losses could reach 20–30% or more depending on levels of carbon pricing and how extreme changes in Douglas-fir forests become (Table 1). Notably, these species do not currently benefit from robust protection through existing protected areas: the median overlap of species’ ranges with protected areas in our study area was 16%, with a maximum of 32% (S1 Table). For most species that gain habitat under the carbon pricing scenarios, the gains are of less magnitude (e.g. 10–13% gains by 2100; Fig 5, Table 1) than the declines observed for other species (additional species-specific maps in S4 and S5 Figs).

**Fig 6:**
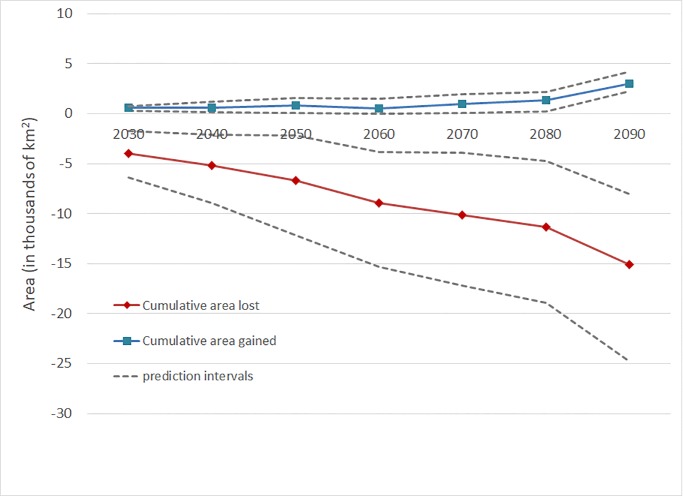
Marginal changes in total habitat area lost and total habitat area gained by decade that arise from climate change and carbon pricing. The y-axis depicts total area of altered habitat (in thousands of km^2^) that occur within each decade (x-axis) beginning in 2030 relative to the baseline. Total areas lost (gained) is computed by summing habitat areas lost (gained) across all 35 wildlife species of conservation concern. Summing the marginal changes across all decades in this graph would generate cumulative habitat changes.

To illustrate the overall change in habitat across all 35 wildlife species of conservation concern, we examined marginal changes in total habitat acreage lost and total habitat acreage gained by decade (Fig 6). In each decade from 2030 to 2100, the total wildlife habitat lost in that decade exceeds the total wildlife habitat gained, and the net loss in habitat per decade accelerates over time. If the ecosystem service price for an acre of wildlife habitat were constant across all species, then Fig 6 could be translated to indicate that the marginal economic damages from lost wildlife habitat exceed the marginal benefits from additional wildlife habitat for the climate change and carbon pricing scenarios considered here. Further, the incremental reductions in wildlife habitat increase as the carbon price is increased, illustrating a direct connection between a carbon mitigation policy and important landscape characteristics that contribute to biodiversity conservation on private forestland.

We only project potential habitat changes within the current range of each species we examined, and so we do not account for future species’ range expansion into areas that may become more climatically suitable, with more suitable forest types. We limited our study to current ranges because of the considerable uncertainty in modeling species’ range shifts [43], especially with regard to constraints on species vagility. However, nothing precludes combining our analysis with range projections to provide an additional assessment of effects on forest wildlife should that be a desirable analysis extension.

## Discussion

Whether private forests will continue to provide the same wildlife habitat under climate change will depend in part on private forest management practices [44,45]. An important avenue by which climate change and climate change mitigation policies will affect biodiversity is through private forest landowners’ adaptation behavior. Additionally, carbon pricing is one of the most widely advocated policy approaches for incentivizing carbon sequestration worldwide [46] and is increasingly likely to play a role in influencing forest management in the future. Our results suggest that landowner adaptation can have multiple impacts on species that are forest habitat specialists. In the U.S. Pacific coast states, landowner responses to climate change shift potential forest habitat away from coniferous Douglas-fir forests and towards hardwood forests, leading to habitat losses for coniferous forest (other than ponderosa pine) specialists and habitat gains for hardwood and ponderosa pine specialists. More wildlife species of conservation concern drawn from state agency lists are projected to lose, rather than gain, potential habitat. We find that carbon pricing would accelerate adaptation away from coniferous forests (excepting pines) and towards hardwoods, pointing to a potential conflict between carbon pricing policy and wildlife conservation policy that aims to preserve existing habitat for species experiencing declines (e.g., U.S. Endangered Species Act). Altogether, the projected total wildlife habitat lost in each future decade exceeds the total wildlife habitat gained, and the net loss in habitat per decade accelerates over time.

Douglas-fir is the most commercially dominant and heavily managed timber species across the study region [47], and the most common forest type in the Pacific Northwest. Hardwoods are mostly regenerated naturally following harvest, tend to be managed less intensively, and tend to be worth less per unit in timber markets. Natural science analyses anticipate that climate change will significantly reduce Douglas-fir productivity in the coastal portion of the Pacific Northwest [2,33,48,49], which is where the majority of commercial value currently resides. Using an alternative analytical method examining private forest landowner behavior [3], our study complements natural science findings that Douglas-fir productivity generally will decline under climate change. Our results suggest that landowners are less likely to replant Douglas-fir in climates that are projected to become warmer and drier than the current climate conditions of northwest Oregon and western Washington. The average productivity of hardwoods is projected to be roughly similar to current levels under climate change. Carbon pricing will favor moving out of forest types that are becoming less productive under climate change (Douglas-fir), and towards regenerating forest types whose sequestration productivity is highest. The projected net habitat losses from carbon pricing in our study arise largely because the forest type expected to experience the greatest losses (Douglas-fir) has supported greater numbers of wildlife species of conservation concern than the forest type that gains (hardwoods).

Our work contributes to the growing body of research by combining an econometric model of private forest management with spatially explicit data characterizing habitat potential for species of policy concern. Recent studies examining climate adaptation in British agriculture found that associated land use and management changes could lower river water quality [50,51]. Other studies have examined the influence of carbon pricing policies on land-use change into and out of forests [21,24,25], but have not explicitly accounted for landowners’ climate change adaptation behaviors, nor modeled changes within existing forests. Although studies have focused on projecting species’ range shifts in response to climatic changes (e.g., via bioclimatic envelope modeling [18–20]), such efforts typically have not considered species-habitat interactions in the context of landowner management. Other methods to assess species’ vulnerability to climate change exist but require more species-specific data than typically available [52].

Our results show that carbon pricing can generate localized costs (externalities) in the form of wildlife habitat losses that result from land-use changes within forestry. When forest types that are most valuable for biodiversity experience climate-induced increases in their carbon sequestration productivity, carbon pricing may generate beneficial changes in wildlife habitat. However, the habitat losses we find result from the fact that the forest type responsible for habitat for more conservation-sensitive wildlife is projected to experience reductions in productivity under climate change. Given current interest in developing carbon pricing policies that include forestry, it may be prudent to examine further the potential tradeoffs that could arise with carbon pricing [53]. There are important tradeoffs to consider when developing ecosystem services pricing policies that apply to only a subset of desired ecosystem services. In the absence of optimal pricing of all ecosystem services, policies which mitigate and encourage forest landowners to adapt to climate change could be developed in concert with wildlife conservation policy to provide for multiple sustainability goals including the provisioning of wood, wildlife habitat, watershed protection, and carbon sequestration (et al.) [54].

Future research could examine spatial interactions between public land and private land when management objectives vary across the landscapes, but habitats extend across management boundaries. Federal policy has called on the US Forest Service to lead efforts to mitigate and adapt to climate change [55], in part, by taking an “all-lands” approach to management that considers roles for both public and private lands [56]. One national forest—the Rogue River-Siskiyou, located in southwestern Oregon—recently proposed an emphasis on legacy species such as oaks and pines over planted Douglas-fir to help these stands better adapt to hotter and drier climates. A question for future research is how such a climate adaptation approach taken on public lands might interact with anticipated climate adaptation on private lands.

The implications of current and projected climate change for forest-dependent biodiversity include substantial uncertainty. The long-term persistence of species affected by climate change will depend on one or more “strategies.” Species can: (1) persist *in situ* where current climate conditions and vegetation types are not expected to change, e.g. in climate refugia [57]; (2) shift their distribution towards more climatically suitable areas, e.g. species’ range shifts [58]; and/or (3) adapt to new climate conditions in either current or shifted geographic ranges, which potentially include “no-analog” climates and communities [59]. Each of these strategies poses substantial challenges: persisting *in situ* requires viability in reduced and potentially isolated ranges; shifting distributions requires landscape-level movements through potentially unsuitable habitats; and adapting to no-analog climates/vegetation types requires either phenotypic plasticity or the genetic diversity that allows for microevolutionary adaptation [60].

Our results suggest that in addition to these challenges, some species that associate with the forest types that are currently dominant on privately owned forests in the Pacific Northwest (namely Douglas-fir) will lose a portion of their potentially suitable habitat, as a result of landowner decisions in response to the very same climate change that the species are facing. These potential losses for some species reach 20–30% or more, especially under high carbon price inputs and at the high end of simulated Douglas-fir losses. Such losses could influence the long-term viability of species already facing challenges, perhaps not successfully, listed above. Particular attention should be placed on species already considered ‘Near Threatened’ (IUCN 2018) that experience the greatest losses in potential habitat under the climate only or carbon pricing scenarios (Fig 5 and S1 Table, see e.g., the Cascade torrent salamander and red tree vole). At the same time, other species experience significant gains (although only 5/35 species) and in the current study we do not consider species that may become of conservation concern in the future that would benefit from expanding hardwood and/or ponderosa pine forest types.

Our finding that one of the principal mitigation policies to increase carbon sequestration (carbon pricing) could exacerbate potential habitat losses is notable and highlights the need to consider the unintended consequences of such policies. All of the typical recommendations to help wildlife species adapt to climate change—increasing habitat connectivity, reducing barriers to movement, restoring ecosystem processes, and considering translocations [61]—still apply, given the changes on private forests we describe. To this list of recommendations, we would add a policy suggestion from the field of environmental economics, which is to consider the value of as many other ecosystem services as possible, including wildlife habitat in general, and/or for species of conservation concern. We acknowledge the difficulty of considering (much less estimating values for) all of the potential use and non-use values of land. However, in order to account for the tradeoffs between benefits and costs—including unintended externalities—from mitigation policy, we need to understand the societal value of wildlife species, which may include significant non-use values [62]. In our study region, there is evidence that non-use values for conserving threatened species can be of substantial magnitude [63]. When considering management actions to shape future landscapes given expected climate change, efficient conservation policy requires that we quantify the costs and benefits associated with such actions [64]. Given the central role of economic activity in causing a rapid change in natural systems, economists and natural scientists should continue to expand collaboration for a sustainable future [65].

## Supporting information

S1 Fig Nested structure of harvest/replanting decisions. (DOCX)

S2 Fig Landscape simulation steps. (DOCX)

S3 Fig Current tree species types and projected climate change. (DOCX)

S4 Fig Effects of climate change, by ecoregion, for selected species. (DOCX)

S5 Fig Combined effects of climate change and carbon prices, by ecoregion for selected species. (DOCX)

S6 Fig Level 3 ecoregions. (DOCX)

S1 Table Forest wildlife species in taxonomic order with conservation status and forest type associations. (DOCX)

S1 Text Econometric framework. (DOCX)

S2 Text Simulation. (DOCX)
