# Supplementary material for: Anticipating changes in wildlife habitat induced by private forest owners’ adaptation to climate change and carbon policy
Source: PLoS One. 2020 Apr 2;15(4):e0230525. doi: 10.1371/journal.pone.0230525 (PMC7117685; doi:10.1371/journal.pone.0230525)
Supplement: S1 Fig — (DOCX) [file pone.0230525.s001.docx]

Figure S1: Nested structure of harvest/replanting decisions


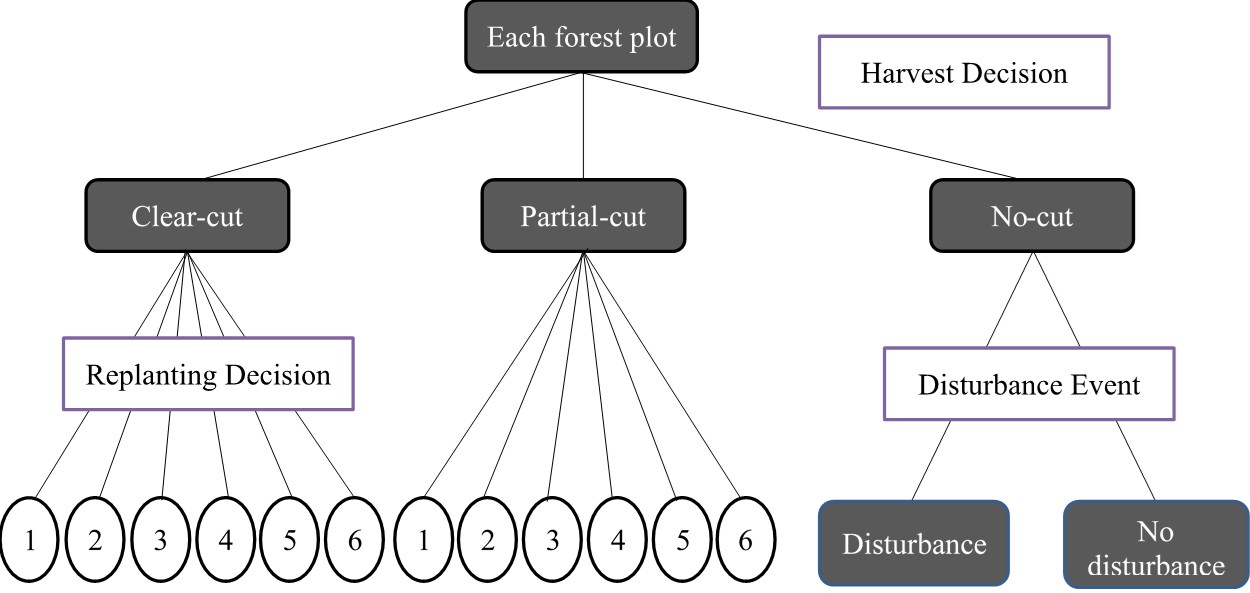


Note: Replanting choices 1 through 6 correspond to Douglas-fir, fir/spruce/mountain hemlock, hemlock/sitka spruce, ponderosa pine, other softwood, and hardwood, respectively. [Figure is adapted from Hashida and Lewis (2019)]
