# Supplementary figures and images for: Anticipating changes in wildlife habitat induced by private forest owners’ adaptation to climate change and carbon policy
Source: PLoS One. 2020 Apr 2;15(4):e0230525. doi: 10.1371/journal.pone.0230525 (PMC7117685; doi:10.1371/journal.pone.0230525)

Figure S2: Landscape simulation steps


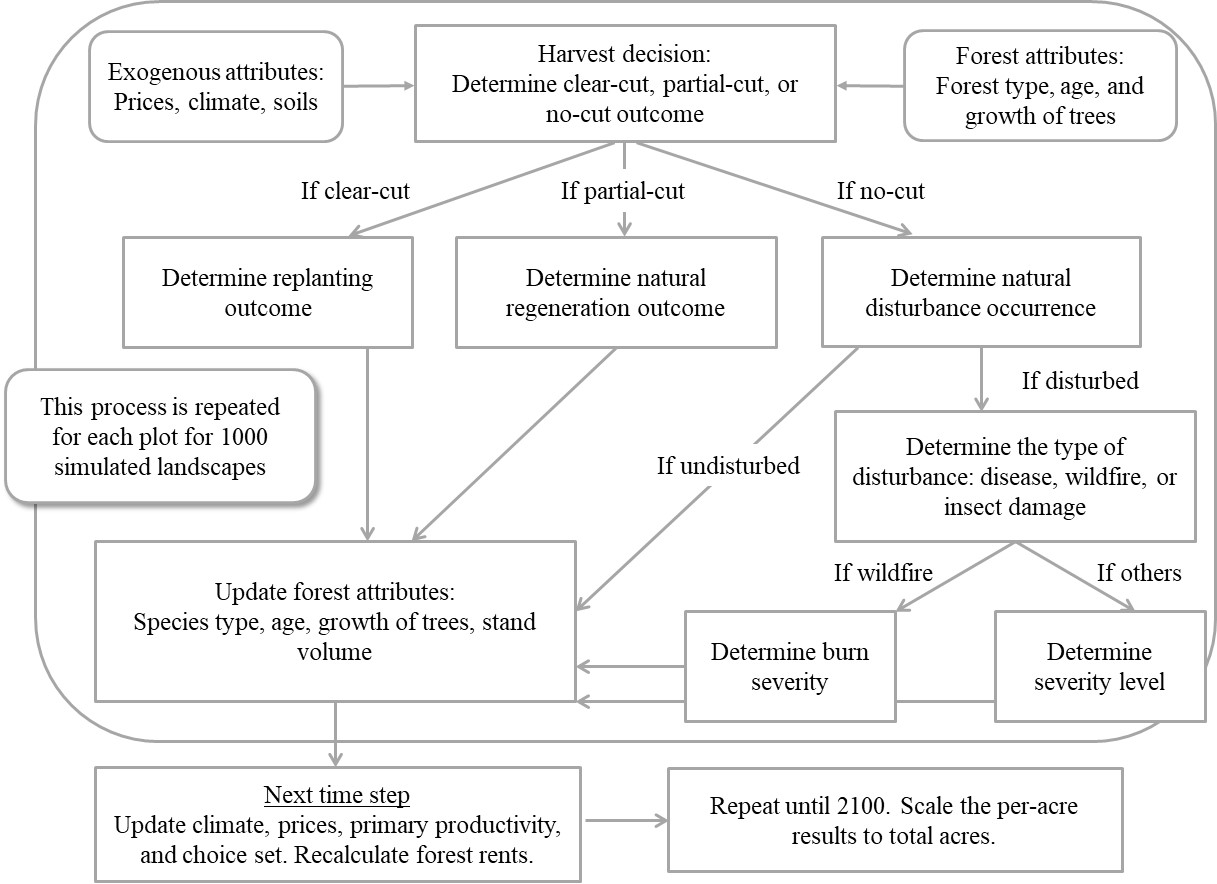


Note: Figure is adapted from Hashida and Lewis (2019)

Supplement: S2 Fig — (DOCX) [file pone.0230525.s002.docx]
