# Supplementary material for: Anticipating changes in wildlife habitat induced by private forest owners’ adaptation to climate change and carbon policy
Source: PLoS One. 2020 Apr 2;15(4):e0230525. doi: 10.1371/journal.pone.0230525 (PMC7117685; doi:10.1371/journal.pone.0230525)
Supplement: S3 Fig — (DOCX) [file pone.0230525.s003.docx]

Figure S3: Current tree species types (left) and projected climate change (right)

Note: “Much warmer” and “Much drier” indicate 4 degrees Celsius or more increase in temperature and larger than 50 mm precipitation decrease.
Source: AdaptWest Project. 2015. Gridded current and projected climate data for North America at 1km resolution, interpolated using the ClimateNA v5.10 software (T. Wang et al., 2015).

*3.b Projected climate change*

*3.a Current distribution of forest types*
