# Supplementary material for: Anticipating changes in wildlife habitat induced by private forest owners’ adaptation to climate change and carbon policy
Source: PLoS One. 2020 Apr 2;15(4):e0230525. doi: 10.1371/journal.pone.0230525 (PMC7117685; doi:10.1371/journal.pone.0230525)
Supplement: S4 Fig — (DOCX) [file pone.0230525.s004.docx]

Figure S4: Effects of climate change, by ecoregion, for selected species (percent potential habitat gained or lost through 2090 under climate change only scenario, relative to baseline). Species’ ranges may only overlap part of an ecoregion, even though the percent change is colored in for the entire ecoregion.

White-footed vole

Dunn's salamander

Southern torrent salamander

Red tree vole

Fisher

Yellow-billed magpie


Pallid bat
