# Supplementary material for: Anticipating changes in wildlife habitat induced by private forest owners’ adaptation to climate change and carbon policy
Source: PLoS One. 2020 Apr 2;15(4):e0230525. doi: 10.1371/journal.pone.0230525 (PMC7117685; doi:10.1371/journal.pone.0230525)
Supplement: S5 Fig — (DOCX) [file pone.0230525.s005.docx]

Figure S5: Combined effects of climate change and carbon prices, by ecoregion for selected species (percent potential habitat gained or lost through 2090 under climate change only scenario, relative to baseline)

Fisher

White-footed vole

Southern torrent salamander

Dunn’s salamander


Red tree vole


Ringtail


Pallid bat
