# Supplementary material for: Anticipating changes in wildlife habitat induced by private forest owners’ adaptation to climate change and carbon policy
Source: PLoS One. 2020 Apr 2;15(4):e0230525. doi: 10.1371/journal.pone.0230525 (PMC7117685; doi:10.1371/journal.pone.0230525)
Supplement: S6 Fig — (DOCX) [file pone.0230525.s006.docx]

Figure S6: Level 3 ecoregions
