# Supplementary material for: Anticipating changes in wildlife habitat induced by private forest owners’ adaptation to climate change and carbon policy
Source: PLoS One. 2020 Apr 2;15(4):e0230525. doi: 10.1371/journal.pone.0230525 (PMC7117685; doi:10.1371/journal.pone.0230525)
Supplement: S1 Table — (DOCX) [file pone.0230525.s007.docx]

Table S1: Forest wildlife species (N = 35) in taxonomic order with conservation status and forest type associations. IUCN status from the Red List of Threatened Species [7] where LC = Least Concern, NT = Near Threatened, and VUL = Vulnerable (in order of increasing extinction risk). U.S. ESA status is the listing status under the U.S. Endangered Species Act as of 30 June 2018 (no text = not listed). The six forest type columns refer to the aggregate FIA forest types used in this study (SI: Econometric Framework), with 1 = used, and 0 = typically not used, for each species. The last two columns represent the percent overlap, calculated for ‘Study area’ (which represents how much of the entire species’ range is within the study area of three states), and ‘Protected areas’ (which is the % overlap of GAP level 1 and 2 protected areas in CA, OR and WA with the species’ range clipped to those three states, i.e. not the entire species’ range).

| Common name | Scientific name | IUCN status | U.S. ESA status | Douglas-fir | Fir/ Spruce | Hem-lock | Ponderosa Pine | Other softwood | Hard-wood | Study area | Protected areas |
| --- | --- | --- | --- | --- | --- | --- | --- | --- | --- | --- | --- |
| **Amphibians** |  |  |  |  |  |  |  |  |  |  |  |
| Cascades frog | *Rana cascadae* | NT | candidate | 1 | 1 | 1 | 0 | 1 | 0 | 100% | 27.1% |
| Oregon slender  salamander | *Batrachoseps wrighti* | VUL | petitioned | 1 | 0 | 1 | 0 | 0 | 1 | 100% | 21.6% |
| Dunn's salamander | *Plethodon dunni* | LC |  | 1 | 1 | 1 | 0 | 0 | 0 | 100% | 7.2% |
| Del Norte salamander | *Plethodon elongatus* | NT |  | 1 | 0 | 0 | 0 | 1 | 0 | 100% | 24.4% |
| Larch Mountain salamander | *Plethodon larselli* | NT |  | 1 | 0 | 0 | 0 | 0 | 0 | 100% | 14.0% |
| Van Dyke's salamander | *Plethodon vandykei* | LC |  | 1 | 1 | 1 | 0 | 0 | 0 | 100% | 22.8% |
| Cascade torrent  salamander | *Rhyacotriton cascadae* | NT | petitioned | 1 | 1 | 1 | 0 | 0 | 0 | 100% | 15.2% |
| Southern torrent  salamander | *Rhyacotriton variegatus* | LC |  | 1 | 1 | 1 | 0 | 1 | 0 | 100% | 15.2% |
| **Birds** |  |  |  |  |  |  |  |  |  |  |  |
| Ruffed grouse | *Bonasa umbellus* | LC |  | 1 | 1 | 1 | 0 | 1 | 1 | 3.1% | 11.8% |
| Sooty grouse | *Dendragapus fuliginosus* | LC |  | 1 | 1 | 1 | 0 | 1 | 0 | 39% | 18.3% |
| Vaux’s swift | *Chaetura vauxi* | LC |  | 1 | 1 | 1 | 0 | 1 | 0 | 23% | 17.8% |
| Rufous hummingbird | *Selasphorus rufus* | LC |  | 1 | 1 | 1 | 0 | 1 | 0 | 16% | 13.2% |
| Lewis’s woodpecker | *Melanerpes lewis* | LC |  | 1 | 0 | 1 | 1 | 1 | 1 | 18% | 19.7% |
| White-headed  woodpecker | *Picoides albolarvatus* | LC |  | 1 | 1 | 0 | 1 | 1 | 0 | 83% | 18.5% |
| Black-backed  woodpecker | *Picoides arcticus* | LC |  | 1 | 1 | 0 | 1 | 1 | 0 | 3.8% | 16.1% |
| Yellow-billed magpie | *Pica nuttalli* | NT |  | 0 | 0 | 0 | 0 | 0 | 1 | 100% | 10.5% |
| Oak titmouse | *Baeolophus inornatus* | LC |  | 0 | 0 | 0 | 1 | 1 | 1 | 89% | 11.9% |
| White-breasted nuthatch | *Sitta carolinensis* | LC |  | 1 | 0 | 0 | 1 | 1 | 1 | 8.0% | 16.0% |
| Chipping sparrow | *Spizella passerina* | LC |  | 1 | 0 | 0 | 1 | 1 | 1 | 5.1% | 17.8% |
| Lawrence's goldfinch | *Spinus lawrencei* | LC |  | 0 | 0 | 0 | 0 | 1 | 1 | 44% | 32.4% |
| **Mammals** |  |  |  |  |  |  |  |  |  |  |  |
|  |  |  |  |  |  |  |  |  |  |  |  |
| White-footed vole | *Arborimus albipes* | LC |  | 1 | 1 | 1 | 1 | 1 | 0 | 100% | 8.5% |
| Red tree vole | *Arborimus longicaudus* | NT | candidate | 1 | 1 | 1 | 0 | 0 | 0 | 100% | 6.9% |
| Sonoma tree vole | *Arborimus pomo* | NT |  | 1 | 1 | 1 | 0 | 0 | 0 | 100% | 13.6% |
| Lodgepole chipmunk | *Neotamias speciosus* | LC |  | 1 | 0 | 0 | 1 | 1 | 0 | 98% | 29.8% |
| Western gray squirrel | *Sciurus griseus* | LC |  | 1 | 0 | 1 | 1 | 1 | 1 | 98% | 14.5% |
| Merriam’s shrew | *Sorex merriami* | LC |  | 1 | 0 | 0 | 1 | 1 | 1 | 14% | 11.6% |
| Pallid bat | *Antrozous pallidus* | LC |  | 0 | 0 | 0 | 1 | 1 | 1 | 20% | 17.3% |
| Western red bat | *Lasiurus blossevillii* | LC |  | 1 | 1 | 1 | 0 | 1 | 1 | 3.0% | 20.4% |
| Long-legged myotis | *Myotis volans* | LC |  | 1 | 1 | 1 | 1 | 1 | 0 | 17% | 17.8% |
| Yuma myotis | *Myotis yumanensis* | LC |  | 1 | 0 | 1 | 1 | 1 | 0 | 21% | 17.8% |
| Ringtail | *Bassariscus astutus* | LC |  | 0 | 0 | 0 | 1 | 1 | 1 | 8.1% | 20.7% |
| Wolverine | *Gulo gulo* | LC | proposed | 1 | 1 | 1 | 1 | 1 | 0 | 0.3% | 14.9% |
| Lynx | *Lynx canadensis* | LC | threatened | 1 | 1 | 1 | 1 | 1 | 0 | 0.9% | 18.9% |
| Fisher | *Pekania pennanti* | LC |  | 1 | 1 | 1 | 0 | 1 | 0 | 7.2% | 15.7% |
| Sierra Nevada red fox | *Vulpes vulpes necator* | LC | candidate | 0 | 1 | 0 | 1 | 1 | 0 | 0.4%^a^ | 12.7% |

^a^ Calculation based on global range of *Vulpes vulpes* to be consistent with other species’ calculations. The range of the subspecies *Vulpes vulpes necator* is entirely (100%) within the three study area states.
