# Supplementary material for: Anticipating changes in wildlife habitat induced by private forest owners’ adaptation to climate change and carbon policy
Source: PLoS One. 2020 Apr 2;15(4):e0230525. doi: 10.1371/journal.pone.0230525 (PMC7117685; doi:10.1371/journal.pone.0230525)
Supplement: S1 Text — (DOCX) [file pone.0230525.s008.docx]

**S1: Econometric Framework**

The econometric framework was originally developed in Hashida and Lewis (2019), and we refer readers to that paper for a more in-depth treatment. In this section we provide a condensed presentation of the econometric framework from Hashida and Lewis (2019). The econometric model is designed to model forest management as a set of discrete choices using contemporary discrete choice methods (see Train 2009) [1,2]. S1 Fig shows a schematic of the decision-making structure of the landowner that we use to specify the econometric model. The first decision a landowner makes is whether to harvest her land as a clear-cut or partial-cut, or not harvest her land and let the trees grow. If the landowner harvests her land, then she chooses whether to replant/re-generate her land in one of six primary forest types that grow in the Pacific Northwest (S1 Fig). If the landowner does not harvest her land, then it either grows to the next period or is affected by a natural disturbance such as wildfire.

Given the decision-making structure in S1 Fig, the econometric model is built on a theoretical foundation developed in natural resource economics (e.g. Guo and Costello 2013). Landowner *n* has a forest stand growing forest type *s* of age *a* and faces a local climate described by the vector *c*. The landowner is assumed to choose management action *j* in time *t* to maximize their land value function $V_{njt}$, which equals:

$V_{njt}(s,a,c_{t})=V_{nkt}^{h}(s,a)+V_{njt}^{ph|h}(s,a,c_{t})+\varepsilon_{njt}$ (S1)

where $V_{njt}^{ph|h}$ is unique to post-harvest outcome *j*, and $V_{nkt}^{h}$ is unique to harvest choice *k* and common to all post-harvest outcomes in $h_{k}$, including *k*. The term $\varepsilon_{njt}$ is observable to the landowner but not the researcher. Management action $j$ is chosen if $V_{njt}>V_{nit}$for all $i\neq j$.

We specify the post-harvest land value function $V_{njt}^{ph|h}$ for the replanting choice (bottom left and center of S1 Fig) as a function of the average per-acre rent (in dollars) for planting species *s_j_* in region *r* that contains plot *n* ($\bar{rent}_{r\left( n \right)s_{j}t}$), a vector of down-scaled climate variables ($c_{nt}$), climate forecasts thirty years into the future ($c_{nt+30}$), and the plot’s elevation (${elev}_{n}$):

$$V_{njt}^{ph|h}=f_{replant}(\bar{rent}_{r\left( n \right)s_{j}t},c_{nt},c_{nt+30},{elev}_{n}; \beta_{j}^{ph})$$

$for ph\in\{replant\left| clearcut, regenerate \right|partial cut\}$ (S2)

where $\beta_{j}^{ph}$ represent a vector of post-harvest parameters to estimate. The climate variables ($c_{nt}$) include the mean monthly temperature during the growing season, total monthly precipitation during the growing season, and the monthly 30-year normal minimum and maximum temperatures.

We also estimate the risk of natural disturbance through the “no-cut” nest (bottom right of Figure S1). Conditional on not harvesting (“no-cut”), the landowner leaves the stand at risk to the binary outcomes of natural disturbance or no disturbance. We jointly estimate drivers of disturbance and harvest decisions by specifying the lower “no cut” nest as a reduced form binary model:

$$V_{njt}^{ph|h}=f_{disturb}({priv}_{n},{elev}_{n},{species}_{nt},{vol}_{nt},c_{nt},{state}_{n}; \beta_{j}^{ph})$$

$for ph\in\{disturbance event|no cut\}$ (S3)

The independent variables that affect the probability of natural disturbance include an ownership dummy indicating private or state ownership (${priv}_{n}$), elevation (${elev}_{n}$), tree species dummy variables indicating the forest type (${species}_{n}$), the current timber volume (${vol}_{nt}$), a state dummy (${state}_{n}$), and a vector of climate variables ($c_{nt}$).

In the upper nest in S1 Fig, the forest landowner chooses whether to clear-cut harvest, partial-cut harvest, or not cut a forest stand. We specify the observable components specific to the net revenue from harvest method *h* as:

$V_{nkt}^{h}=g(P_{ns_{j}t}\cdot{vol}_{nk\left( s_{j} \right)t}^{h};\alpha_{k})$ (S4)

$$for h\in\{clear cut, partial cut\}$$

Where $P_{ns_{j}t}$ is the timber price that landowner *n* faces for forest type $s_{j}$ in time *t*, and ${vol}_{nk\left( s_{j} \right)t}^{h}$ is the volume of timber that landowner *n* can harvest using method *h*. By interpreting $\alpha_{0k}$ as an expected harvest cost, then $V_{nkt}^{h}$ approximates the net one-period profit (marginal benefit) that landowner *n* could extract from harvesting with method *h* and selling the trees. We specify the observable components specific to the decision not to cut and let the stand grow as a function of the expected changes in revenue from volume growth:

$V_{nkt}^{h}=g(P_{ns_{j}t}\cdot{\Delta vol}_{nk\left( s_{j} \right)t};\alpha_{k})$ (S5)

$$for h\in\{no cut\}$$

where ${\Delta vol}_{nk(s_{j})t}$ represents the incremental volume growth^[[1]](#footnote-1)^ that landowner *n* would generate from letting the trees grow. Multiplying ${\Delta vol}_{nk(s_{j})t}$ by price $P_{ns_{j}t}$ represents the growth in revenue from not cutting (a marginal cost of harvesting). Since clear cut harvests generate more volume than partial cutting^[[2]](#footnote-2)^, the volume variable is indexed by harvest choice *k*. Hashida and Lewis (2019) jointly estimate all parameters in (S2) – (S5) using maximum likelihood techniques, and we refer readers to that paper for parameter estimates and more detail on data construction.

Train (2009) shows how this type of discrete-choice maximization problem generates an estimable probability that landowner *n* chooses management action *j* in time *t* as a product of the probability of harvest action *k* multiplied by the probability of post-harvest outcome *j* conditional on choosing harvest *k*:

${Prob}_{njt}(V_{njt}^{ph|h},V_{nkt}^{h})={Prob}_{nkt}\cdot{Prob}_{njt|k}$ (S6)

The econometric model generates a plot-level probability that a landowner harvests a forest land in clear-cut or partial-cut or leaves the stand undisturbed to grow. Conditional on the landowner harvesting the land, the econometric model also generates a plot-level probability of the forest type that the landowner chooses to replant on the land. Conditional on the landowner not harvesting the land, the econometric model generates a plot-level probability that the plot is naturally disturbed.

1. Section S6 has a detailed description of how tree growth is calculated for each plot. [↑](#footnote-ref-1)
2. Partial cut volume is estimated by comparing the measured volumes in 10-year intervals for the re-measured plots that have a record of partial cut treatment in the most recent survey. For the other plots, we assigned the percentage of partial cut portion of total volume according to the available information such as the treatment code that distinguishes “less than 20% removed” or “more than 20% removed”, as well as county average percentage across the re-measured partially-cut plots. [↑](#footnote-ref-2)
