# Supplementary material for: Anticipating changes in wildlife habitat induced by private forest owners’ adaptation to climate change and carbon policy
Source: PLoS One. 2020 Apr 2;15(4):e0230525. doi: 10.1371/journal.pone.0230525 (PMC7117685; doi:10.1371/journal.pone.0230525)
Supplement: S2 Text — (DOCX) [file pone.0230525.s009.docx]

**S2: Simulation**

We use our econometrically estimated forest management and natural disturbance probabilities as a set of decision rules to simulate forest management changes on each plot, and then aggregate across plots to simulate changes in the landscape of forest types and corresponding changes in wildlife habitat for the thirty-five species of conservation concern under a changing climate and a carbon price policy. While we use Hashida and Lewis’ (2019) simulations of future landscape forest types, this analysis differs by translating the future forest types to potential wildlife habitat. Beginning with the current composition of forest stock across the landscape, the simulation uses the econometric estimates to adjust plot-level management probabilities to exogenous changes in climate as well as corresponding and previously estimated climate change effects on global timber prices [3] and the net primary productivity of forests [4]. The simulation then generates endogenous changes in the forest stock, including the timing and intensity of harvest, natural disturbance, the composition of different forest types, and the composition of potential wildlife habitat for thirty-five species in repeated 10-year intervals until the year 2100. S2 Fig presents a schematic of the simulation.

Future climate regimes are derived from the IPCC’s Fifth Assessment Report (AR5). Monthly temperature and precipitation output were based on the results from the U.S. National Center for Atmospheric Research (NCAR) Community Climate System Model (CCSM) 4. The downscaled data at 1km resolution was obtained from the ClimateWNA model developed by the Center for Forest Conservation Genetics at the University of British Columbia [5]. S3b Fig is a descriptive map that highlights an overall projected climate trend of temperature and precipitation for each FIA plot. The area shown in brown, the majority of premier private forestland in the west side of the Cascades, is expected to become warmer and drier. Under the RCP 8.5 scenario, average temperature is expected to increase by 4.35 ºC by 2100 in the study region. We use Hashida and Lewis’ (2019) simulation of a hypothetical carbon pricing scheme that starts in 2020 and in which landowners receive fixed payments for the amount of carbon sequestered in their forests. Consistent with contemporary ideas about an increasing carbon price[6], we assume that a carbon price starts at $15/ton in 2020, rises to $50 in 2050, and again to $80 in 2080^[[1]](#footnote-1)^.

1. As of March 29, 2018, carbon price in California Carbon Allowance Futures is around $15.10/tonne CO2 equivalent. [↑](#footnote-ref-1)
